# Small Segmental Duplications in *Drosophila*—High Rate of Emergence and Elimination

**DOI:** 10.1093/gbe/evz011

**Published:** 2019-01-28

**Authors:** Juan Li, Lan Jiang, Chung-I Wu, Xuemei Lu, Shu Fang, Chau-Ti Ting

**Affiliations:** 1 Key Laboratory of Genomics and Precision Medicine, Beijing Institute of Genomics, Beijing; CAS Center for Excellence in Animal Evolution and Genetics, Kunming Institute of Zoology, Kunming, Chinese Academy of Sciences, China; 2 University of Chinese Academy of Sciences, Beijing, China; 3 Institute of Ecology and Evolutionary Biology, National Taiwan University, Taipei, Taiwan; 4 Department of Ecology and Evolution, University of Chicago; 5 School of Life Science, Sun Yat-Sen University, Guangzhou, China; 6 Biodiversity Research Center, Academia Sinica, Taipei, Taiwan; 7 Department of Life Science, Center for Biotechnology, Center for Developmental Biology and Regenerative Medicine, National Taiwan University; 8 Genome and Systems Biology Degree Program, National Taiwan University and Academia Sinica, Taipei, Taiwan

**Keywords:** haploid genome, isogenic genome, polymorphic duplication, pseudoheterozygous site, purifying selection

## Abstract

Segmental duplications are an important class of mutations. Because a large proportion of segmental duplications may often be strongly deleterious, high frequency or fixed segmental duplications may represent only a tiny fraction of the mutational input. To understand the emergence and elimination of segmental duplications, we survey polymorphic duplications, including tandem and interspersed duplications, in natural populations of *Drosophila* by haploid embryo genomes. As haploid embryos are not expected to be heterozygous, the genome, sites of heterozygosity (referred to as pseudoheterozygous sites [PHS]), may likely represent recent duplications that have acquired new mutations. Among the 29 genomes of *Drosophila melanogaster*, we identify 2,282 polymorphic PHS duplications (linked PHS regions) in total or 154 PHS duplications per genome. Most PHS duplications are small (83.4% < 500 bp), *Drosophila melanogaster* lineage specific, and strain specific (72.6% singletons). The excess of the observed singleton PHS duplications deviates significantly from the neutral expectation, suggesting that most PHS duplications are strongly deleterious. In addition, these small segmental duplications are not evenly distributed in genomic regions and less common in noncoding functional element regions. The underrepresentation in RNA polymerase II binding sites and regions with active histone modifications is correlated with ages of duplications. In conclusion, small segmental duplications occur frequently in *Drosophila* but rapidly eliminated by natural selection.

## Introduction

Gene duplications play an important role in phenotypic divergence and evolutionary innovation (Ohno 1970). Extensive studies have been mainly focused on the rates of evolution (Ohta 1980), evolutionary fates (Force et al. 1999; Lynch and Conery 2000), phenotypic diversity (e.g., Hox gene family, Ruddle et al. 1994), functional divergence (reviewed by Conant and Wolfe [2008]), and the mechanisms of gene duplication (reviewed by Hastings et al. [2009]). Several models propose the way in which duplicated genes are preserved in the genomes, such as the duplication–degeneration–complementation model (Force et al. 1999; reviewed by Innan and Kondrashov [2010]). Newly duplicated genes tend to be functionally redundant, and thus one duplicated copy is most likely to be eliminated or silenced by accumulated random mutations. Over time, genetic drift and selection may cause a small proportion of duplicated genes to be fixed and maintained, mainly by subfunctionalization or neofunctionalization.

Classical studies of gene duplication evolution have concentrated on the fixed duplicate genes between species. With accumulation of population genomics studies, investigations on the polymorphic duplicated genes have revealed many features of duplications in the early evolution of gene duplications. Segmental duplications in primate genomes are generally referred to the duplications ranging from 1 kb to several hundreds kb (Eichler 2001; Sharp et al. 2005). However, segmental duplication sizes in *Drosophila melanogaster* are much smaller; they range from 346 to 81.1 kb in length, and a large portion of them have a size <1 kb with only 7.21% of them >10 kb (Fiston-Lavier et al. 2007). Other studies in *Drosophila* showed that majority of duplications are <500 bp in length (Emerson et al. 2008; Rogers et al. 2014) and lineage specific (Emerson et al. 2008; Rogers et al. 2014, 2015). These small segmental duplications are important genetic variation. Yet, they are often strongly deleterious and thus are kept at very low frequency by purifying selection (Dopman and Hartl 2007; Emerson et al. 2008; Cridland and Thornton 2010; Cardoso-Moreira et al. 2011; Langley et al. 2012; Schrider et al. 2013; Rogers et al. 2014, 2015). Purifying selection has also played an important role in shaping the locations of duplicated genes (Dopman and Hartl 2007; Emerson et al. 2008; Cridland and Thornton 2010; Cardoso-Moreira et al. 2011, 2016; Zichner et al. 2013; Rogers et al. 2014). Nevertheless, positive selection has driven some gene duplications—including genes that function in toxin response, immune response against bacteria, mating behavior, olfactory response, oogenesis, and sperm development—to fixation or close to fixation (Emerson et al. 2008; Rogers et al. 2014, 2015; Cardoso-Moreira et al. 2016). During the polymorphic phase, reduction of gene expression has been observed in many whole (or complete) gene duplications, which might result from either loss of cis-regulatory elements in one copy of the duplication or compensation/buffering effects (Cardoso-Moreira et al. 2016; Rogers et al. 2017). New duplications associated with regulatory novelty involved in chimeric structures, untranslated region (UTR) shuffling, or recruitment of noncoding sequence might result in expression-level differences or tissue-specific expression (Rogers et al. 2017).

Previous studies have successfully identified polymorphic retrogenes and tandem duplications with precise breakpoints in *Drosophila* (e.g., Schrider et al. 2011; Zichner et al. 2013; Huang et al. 2014; Rogers et al. 2014, 2015, 2017; Cardoso-Moreira et al. 2016; Tan et al. 2016). However, low copy–interspersed duplications are not easily identified from short read–sequencing data of diploid genomes. Recent studies have shown that heterozygosity observed in haploid genomes, which are not expected to have any true heterozygosity, may result from substitutions between paralogous copies and were thus referred as pseudoheterozygosity (Langley et al. 2011; Pool et al. 2012; Lack et al. 2015). Such pseudoheterozygous sites (PHS), also referred to as “heterozygous” single nucleotide polymorphisms, in hemizygous or inbred lines have also been reported and confirmed in gene duplication studies, for example, *Bar* (Miller et al. 2016) and *rdl* (Remnant et al. 2013). With PHS occurring between small segmental duplications, haploid genomes can provide an independent approach to identify different types of polymorphic duplications including interspersed duplications and to investigate small segmental duplications at the early phase of evolution.

In this study, to understand the emergence of small segmental duplications, we developed a pipeline to identify PHS from haploid genomes and assigned regions with linked PHS as candidate duplications. Those candidate duplications were further validated by in silico and experimental approaches. To study the elimination of small segmental duplications, we characterized the distribution of these polymorphic duplications in different genomic regions. Our analyses of these polymorphic duplications revealed how evolutionary forces shaped the pattern of segmental duplications at the early stage of evolution.

## Results

### PHS Identified from Haploid Genomes Are Associated with Duplications

To identify PHS from the haploid genome of *D.melanogaster*, we established a pipeline with several filtering criteria to remove potential sequencing and mapping errors (see Materials and Methods and supplementary fig. S1, Supplementary Material online). We evaluated this pipeline by analyzing one resequenced genome from the reference strain, *y*; *cn bw sp* (iso-1). This genome was expected to have extremely few heterozygous sites in unique (nonrepetitive) regions because it was sequenced from the same strain as the reference genome. As expected, only four PHS were identified. Using this pipeline, 28 randomly chosen haploid embryo genomes from DPGP2 and 1 autosome-isogenic genome were analyzed (supplementary table S1, Supplementary Material online). In total, 8,253 PHS were identified. Of which, 1,751 clustered in three large duplication regions (>28 kb), one on the chromosome arm 2R and two on the chromosome arm 3R (supplementary table S2, Supplementary Material online), were excluded and the remaining 6,502 PHS were applied in the subsequent analysis because we only focused on small duplications in this study. The average number of PHS per haploid genome was 467 ± 114, ranging from 245 to 739 (supplementary tables S3 and S4, Supplementary Material online). The difference between the resequenced reference and the 29 genomes indicated that this pipeline can effectively identify the PHS from haploid genomes with a low false positive rate (supplementary text, Supplementary Material online).

Among the 6,502 PHS, 72.8% (4,733) were singletons (i.e., present in only one haploid genome), 11.2% (727) were doubletons, and the remaining 16.0% (1,042) were present in at least three genomes. In comparison to the expectation under the neutral model (Watterson 1975), the singleton number is significantly higher than expected, whereas the nonsingleton number is significantly lower than expected (supplementary fig. S2*A*, Supplementary Material online). In addition, the distribution of the PHS per 1-kb nonoverlapping window on chromosome arms showed that these PHS were not evenly distributed (supplementary fig. S3, Supplementary Material online). We further checked the distance between the two closest PHS and found that 77.9% were within 400 bp (supplementary fig. S4, Supplementary Material online). If these PHS were in the regions marked as one copy in the reference genome but had more than one copy in the haploid genomes due to duplications, PHS would therefore represent divergent sites between duplicated copies.

**Fig. 1.**
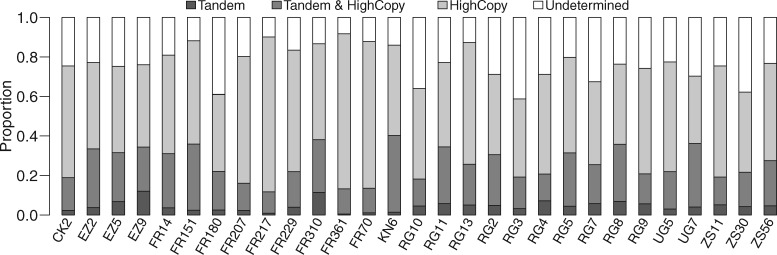
—Percentages of PHS located in different types of duplications. High-copy regions (HighCopy), potential duplications called by read-depth method; tandem duplications (Tandem), duplications called by split-read method; Tandem & HighCopy, duplications called by both read-depth and split-read methods; and undetermined, PHS are not located in the above duplications.

**Fig. 2.**
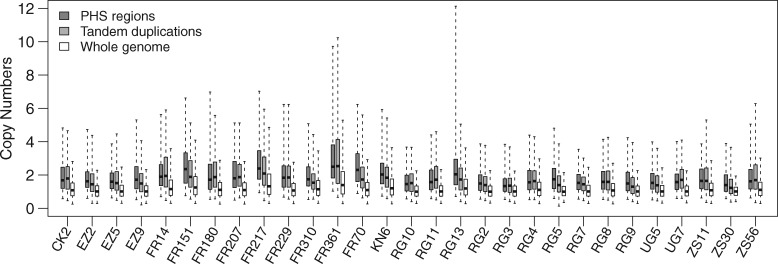
—Average copy numbers of regions with PHS, tandem duplications, and entire genomes. Strain names are labeled on the *x* axis. Copy numbers were predicted by the read-depth method. The median copy numbers are indicated by the central line in a box; the edges of the box represent the first and third quartiles; the whiskers are drawn at the 2.5% and 97.5% quantiles.

To elucidate the relationship between PHS and duplications, we took two independent computational approaches, split read and read depth, to identify tandem duplications and high-copy regions from the haploid genomes, respectively (see Materials and Methods). Using split-read method, we identified 1,920 tandem duplications (supplementary table S5, Supplementary Material online) and found that 25.9% of PHS were located in tandem duplications (fig. 1; supplementary table S4 and supplementary fig. S5, Supplementary Material online). In parallel, the copy numbers across the entire genome were estimated by read depth. The copy numbers of the regions with PHS were significantly higher than that of the entire genome (fig. 2; supplementary table S6, Supplementary Material online). Around 72.4% of PHS were located in high-copy regions, including 21.5% also mapped to tandem duplications, suggesting that a majority [∼70% = 1 − (21.5%/72.4%)] of mapped PHS were located in nontandem duplications, that is, interspersed duplications (fig. 2 and supplementary table S4 and supplementary fig. S5*A*, Supplementary Material online). Together, 76.8% of PHS were located in either tandem duplications or high-copy regions. The remaining 23.2% of the PHS were not mapped into either of the two duplicated regions. We found that the proportion of singleton PHS was higher in undetermined PHS than in silico validated PHS (undetermined PHS: 80.4%, 1,208/1,503 vs. in silico validated PHS: 70.5%, [4,733 − 1,208]/[6,502 − 1,503], Fisher’s exact test, *P = *8.46 × 10^−16^). If the in silico validated PHS are taken as true positives, the higher proportion of singletons in the undetermined PHS might be likely to be false positives. Thus, we inferred that only a small proportion [2.3%, 1,503 × (80.4% − 70.5%)/6,502] of PHS were false positives. The proportion (approximately one quarter) of the undetermined PHS was similar to the proportion of tandem duplications falsely predicted as single copy by read-depth method (fig. 2), supporting that most of the undetermined PHS were located in the high-copy regions with underestimated copy number.

We further validated the copy numbers of 20 PHS by real-time quantitative polymerase chain reaction (qPCR) for two strains to examine the associations between heterozygous sites and duplications. The result showed that 77.5% (=31/20 × 2 strains, supplementary fig. S6, Supplementary Material online) of these PHS were located in the region with copy number significantly >1. Among the nine samples that failed to validate in duplications by qPCR, eight were indicated in duplicated regions by the read-depth and split methods. The PHS that failed to validate might be false positives or within a polymorphic duplicated region, or the primers may have failed to amplify one of the duplications owing to the priming sites across the breakpoints of duplications. By using in silico methods, we found that 31 of the 40 PHS samples (77.5%) were located in high-copy regions or tandem duplications. The ratio based on the 20 PHS data set was similar to that based on the full data set (i.e., 76.8%). Among the nine samples not indicated in duplications by in silico methods, eight were identified to be in duplications by qPCR. Taken together, most PHS are likely in duplicated regions.

For all subsequent analyses, we assigned the duplications based on the existence of the PHS as PHS duplications. Without knowing the breakpoints of duplications, we counted the numbers of duplications inferred in the genome by considering merging linked PHS within 400 bp as one duplication (see Materials and Methods). In total, 2,282 PHS duplications were identified (supplementary tables S7 and S8, Supplementary Material online) and the PHS duplications tended to be accumulated in pericentromeric regions, especially on the chromosome arm 2R (supplementary fig. S7, Supplementary Material online). The average number of PHS duplications per haploid genome was 154 ± 50, ranging from 95 to 364 (supplementary table S4, Supplementary Material online). Most of PHS duplications were small segmental duplications; 83.4% were shorter than 500 bp, 11.3% were between 500 bp and 1 kb, and 5.3% were longer than 1 kb (supplementary table S8, Supplementary Material online). More than half (62.3%, 1,422/2,282) of the PHS duplications overlapped high-copy regions, 21.0% (480/2,282) overlapped tandem duplications (including 15.9% (362/2,282) also overlapping both high-copy regions and tandem duplications), and 32.5% (742/2,282) were left undetermined because they neither overlapped high-copy regions nor tandem duplications (supplementary table S4, and supplementary fig. S5*B*, Supplementary Material online). The ratios of singletons in undetermined PHS duplications were significantly higher than that in the in silico validated PHS duplications [79.9%, 593/742, vs. 69.0%, (1,656 − 593)/(2,282 − 742), Fisher’s exact test, *P = *3.32 × 10^−8^]. If the in silico validated PHS duplications are taken as true positives, we inferred that a small proportion [3.5%, 742 × (79.9% − 69.0%)/2,282] of PHS duplications might be false positive.

A majority (72.6%, 1,656/2,282) of PHS duplications were singletons (supplementary table S8, Supplementary Material online), concordant with results from previously studies in *Drosophila* (Dopman and Hartl 2007; Emerson et al. 2008; Cridland and Thornton 2010; Cardoso-Moreira et al. 2011; Langley et al. 2012; Schrider et al. 2013; Rogers et al. 2014, 2015). Based on the neutral expectation (Watterson 1975), we would expect to observe 581 [=2,282/(1 + 1/2 + 1/3 + … + 1/28)] singleton PHS duplications and 1,701 nonsingleton duplications in the 29 genomes with 2,282 PHS duplications in total. Thus, there was a significant excess of the observed singleton PHS duplications (*χ*^2^ test, *P *<* *10^−15^; supplementary fig. S2*B*, Supplementary Material online). This result suggested that most PHS duplications were deleterious and thus cannot be accumulated in the population. A similar pattern of high elimination rate can also be observed in tandem duplications (*χ*^2^ test, *P *<* *10^−15^, supplementary fig. S2*C*, Supplementary Material online).

### PHS Were Mostly Contributed by Divergence after Duplication

We addressed the origin of these heterozygous sites by investigating if any of these PHS duplications arose in the ancestral lineage leading to *D. melanogaster* and *Drosophilasimulans*. We searched the *D. simulans* homologs of these PHS duplications by standard BlastN with 90% identity in >50% of the PHS duplication length alignable. Among 2,282 PHS duplications, 50.2% (1,146/2,282) had only one copy in *D. simulans*, 47.9% (1,092/2,282) had no homologous sequences in *D. simulans*, and <2% (44/2,282) were found in both species. The results suggested that most of the PHS duplications were lineage-specific duplications. The observed ratio (47.9%) of PHS duplications that have no homologous region in *D. simulans* was significantly higher than the expected 27.8% based on the randomly selected 400-bp genomic fragments (Permutation test, *P *=* *0.001), suggesting that PHS duplications were more likely to occur in highly diverged regions.

We further asked if the sequence difference between two duplicated copies originated from ancestral polymorphism before duplication or from mutations accumulated after duplication. If most of the divergence accumulated before duplication, we would expect to observe similar levels of divergence for PHS duplications in different frequencies. Alternatively, if most of the divergence accumulated after duplication, we would expect to observe less divergence in young duplications that segregated mainly at low frequencies and more divergence in older duplications that segregated mainly at high frequencies. To distinguish these two possibilities, we first compared the proportions of tandem duplications with divergence between singleton versus nonsingleton groups (i.e., young vs. older groups). Here, tandem duplications rather than PHS duplications were analyzed because all PHS duplications were with divergence, whereas tandem duplications were either with or without divergence. It is worth to note that the tandem duplications with divergence were also PHS duplications (hereafter, tandem PHS duplications). The results showed that the proportion of tandem PHS duplications was 15.4% (230/1,492) in the singleton group but 30.4% (130/428) in the nonsingleton group. In other words, the number of tandem PHS duplications was overrepresented in the nonsingleton group (Fisher’s exact test, *P *<* *0.001), which is consistent with little divergences in younger duplications at low frequency and more divergence in older duplications at higher frequency. We then plotted the divergence distribution of both all tandem duplications and the tandem PHS duplications (supplementary fig. S8, Supplementary Material online). The results showed that the majority of these singleton tandem duplications exhibited little divergence, whereas nonsingletons tended to increase divergence as frequency increased. The correlation between tandem PHS duplication and duplication frequencies suggested that PHS were mostly contributed by mutations accumulated after duplication. Assuming most tandem duplications were not false positives, the fact that most tandem duplications have no divergence (81.3%, 1,560/1,920) also support the hypothesis that divergences accumulated during postduplication era.

### Nonrandom Distribution of PHS in Genomic Regions

We investigated the potential effects of duplications with divergence in different genomic regions by examining the distribution pattern of these sites in five genomic regions: coding sequence (CDS) regions, 5′UTR, 3′UTR, introns, and intergenic regions. The number of PHS in CDS and 3′UTR regions was significantly higher than expected, but that in 5′UTR and intronic regions was significantly lower than expected (supplementary table S9, Supplementary Material online; *χ*^2^ test, *P *<* *0.001). No significant difference was found in intergenic regions (*χ*^2^ test, *P *=* *0.49). The overrepresentation of PHS in CDS regions, the underrepresentation of PHS in 5′UTR and introns was clearer in nonsingleton PHS.

The overrepresentation of PHS in specific genomic regions indicated that the evolutionary rate was accelerated in those regions. To examine the accelerated evolution in CDS regions, we compared the nonsynonymous divergence versus synonymous divergence (*d*_N_/*d*_S_) between tandem duplicates that overlapped coding regions and between complete genes that overlapped PHS duplications to understand the driving force in shaping the overrepresentation of heterozygous sites in CDS regions. Among 1,920 tandem duplications, 610 overlapped the coding regions of 753 genes. Out of these 753 genes, 140, including 82 partial and 58 complete gene duplications, accumulated PHS in CDS regions. In addition, 24 complete gene duplications based on the criterion that the entire genic region was within linked heterozygous sites were identified. Among those 24 complete gene duplications, 12 were also identified as tandem duplications, and 10 of the other 12 interspersed genes accumulated divergence in CDS regions. Thus, 150 PHS duplications (140 tandem duplications and 10 interspersed whole-gene duplications) were available to compare *d*_N_/*d*_S_ between paralogous copies (supplementary table S10, Supplementary Material online). Among them, 79 (52.7%) showed accelerated protein evolution rates, including 7 genes with 1 > *d*_N_/*d*_S_ > 0.5, 15 genes with *d*_N_/*d*_S_ > 1, and 57 genes with *d*_N_ > 0 and *d*_S_ = 0. There was no difference in nonsynonymous and synonymous changes between partial gene and complete gene duplications (Wilcoxon rank sum test, *P = *0.291). We also found that the *d*_N_/*d*_S_ ratios between paralogs in *D. melanogaster* were greater than those of orthologs between *D. melanogaster* and *D. simulans* (Sign test, *P *=* *0.0005), suggesting that paralogs evolved more rapidly than orthologs at the early evolution of duplication.

### Duplications Occurred Less Frequently at Active Functional Elements due to Negative Selection

We elucidated the impacts of PHS duplications on functional elements in noncoding regions by examining if the distributions of PHS duplications associated with different genomic functional elements deviated from the expected random genomic regions. Using the functional elements annotated in *Drosophila* modENCODE, we found that a majority of genomic functional elements duplicated less frequently than expectation (supplementary table S11, Supplementary Material online; 60.6% of data sets with permutation test, *P *<* *0.05). These regulatory regions were mainly associated with active functional elements, including active histone modification marks, RNA polymerase II (Pol II), histone deacetylases, and cAMP response element-binding protein regions. In contrast, only a few regulatory regions duplicated more frequently than expected (5.9% of data sets with permutation test, *P *<* *0.05). They were mainly associated with silent functional elements, including heterochromatin protein 1, and methylation at histone lysine 9 (H3K9me3). Therefore, PHS duplications were unevenly distributed at active and silent functional elements.

**Fig. 3.**
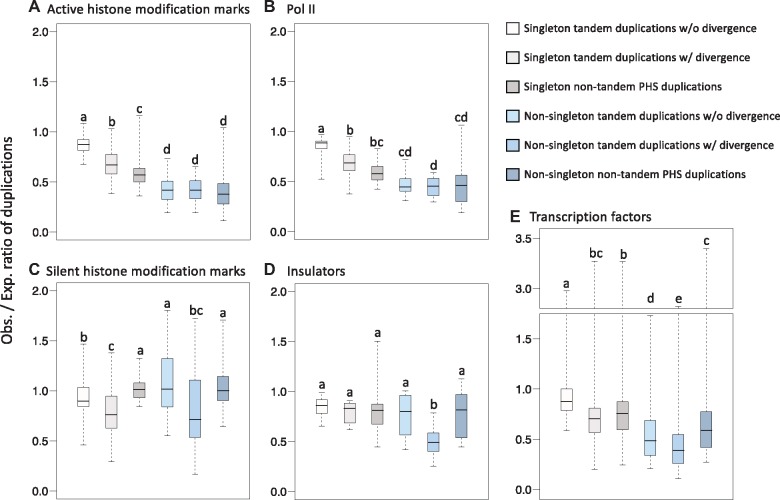
—Distribution of the ratios of observed numbers/expected numbers of duplications at different functional elements. (*A*) Active histone modification marks, (*B*) Pol II, (*C*) silent histone modification marks, (*D*) insulators, and (*E*) transcription factors. Duplications are divided into young singleton duplications (singleton tandem duplications without divergence), old singleton duplications (singleton tandem duplications with divergence and singleton nontandem PHS duplications), and nonsingleton duplications. Different lowercase letters above bars indicate statistically significant differences between duplications groups. Statistical significance was determined by Friedman test followed by multiple comparisons between duplication groups by Wilcoxon signed rank test with Bonferroni correction (*P *<* *0.05, the detailed statistical *P* values are shown in supplementary table S13, Supplementary Material online).

There are two possible explanations to interpret this uneven pattern. Duplication occurred less frequently at active functional elements, or duplication occurred randomly but most duplications at active functional elements were deleterious and were removed over time by natural selection. If duplications occurred nonrandomly, we would expect to see a difference in the number of young duplications between active and silent functional elements. Alternatively, if duplications occurred randomly but are eliminated preferentially by selection, we would expect to observe similar numbers of young duplications between active and silent functional elements but not for older duplications. To distinguish between these two explanations, we compared the differences among six duplication groups with different ages. They can be further combined into three larger groups in an age-ascending order: young singleton duplications (singleton tandem duplications without divergence), old singleton duplications (singleton tandem duplications with divergence and singleton nontandem PHS duplications), and nonsingleton duplications. The deviation from expectation of duplications in each functional element class was calculated as the ratio of the observed number of duplicated functional elements over the expected number. In active functional elements (active histone modification marks and Pol II), all six groups of duplications were significantly lower than expectation (Sign test, *P *<* *0.01). The levels lowering than expectation were correlated with the age of duplications. In general, the level lowering in order was the nonsingleton duplications > the old singleton duplications > the young single duplications (fig. 3*A* and *B* and supplementary tables S12 and S13, Supplementary Material online). In contrast, in silent functional elements (silent histone modification marks and insulators), there was no correlation of duplication reduction level over time (fig. 3*C* and *D* and supplementary tables S12 and S13, Supplementary Material online). In silent histone modification marks, some older groups showed a lower level than the younger groups did but some showed a higher level (fig. 3*C* and supplementary tables S12 and S13, Supplementary Material online). In insulators, the duplication reduction was almost the same in different age groups of duplications (fig. 3*D* and supplementary tables S12 and S13, Supplementary Material online). In transcription factors which were involved in both active and silent functions, the trend was similar to that in active functional elements perhaps because active functions had a larger contribution than silent functions in transcription factors (fig. 3*E* and supplementary tables S12 and S13, Supplementary Material online). These results were in accordance with the explanation that duplications occurred randomly but more duplications at active functional elements were removed over time. We also observed that the level of DNA polymorphism (π and θ) at active functional elements was lower than that in silent functional elements (supplementary table S14, Supplementary Material online). Taken together, these results suggested that most mutations at active functional elements were influenced by negative selection.

## Discussion

To understand the early evolution of duplications within species, we used *D.melanogaster* populations with haploid genome sequences, in which the PHS observed were mostly substitutions between recent paralogous copies. Our results showed that a majority of PHS duplications were small segmental duplications (<500 bp), and interspersed duplications were more abundant than tandem duplications. Overrepresentation of singleton segmental duplications suggested that most of them were deleterious and eliminated by purifying selection. The effect of purifying selection on active functional elements was stronger than on other genomic regions.

PHS in haploid genomes used in this study can result from at least three different factors (Langley et al. 2011; Pool et al. 2012; Lack et al. 2015). First, the haploid genomes used in this study were collected from the maternal genomes of haploid embryos fathered by *ms(3)K81* mutants, rare residual paternal chromosome fragments during gynogenetic development might cause partial diploidy in some cells and result in heterozygosity (Langley et al. 2011, detected in 1 chromosome out of 150 haploid genomes). To exclude this possibility, the genome detected with partial diploidy was not included in our study. Second, heterozygous sites might be caused by somatic mutations. A haploid genome is generated from a single embryo that has undergone fewer than 15 cell divisions (Gilbert 2000), and the observed somatic mutations mainly occur during the first several cell divisions. Given that the estimated somatic mutation rate is <1.3 × 10^−9^ per nucleotide site per cell division in *D. melanogaster* (Lynch 2010), no >3 (<15 cell divisions × 1.3 × 10^−9 ^bp/cell division × 1.1 × 10^8^ bp) heterozygous sites are expected to be observed. Therefore, somatic mutations contribute little to the PHS. Third, mutations accumulated between paralogs might be mistaken for heterozygous sites when the duplications do not exist in the reference genome. When genomic sequences were generated from diploid organisms, this type of PHS cannot be distinguished from single nucleotide polymorphisms, resulting in overestimated heterozygosity (Estivill et al. 2002; Fredman et al. 2004; Cao et al. 2011; Ho et al. 2011). Nevertheless, for genomic sequences generated from haploid organisms, such as those of DPGP, all heterozygous sites were recognized as “pseudoheterozygosity” and masked during data processing (e.g., Lack et al. 2015). Such PHS have been suspected to be found where there is copy number variation (Pool et al 2012; Remnant et al. 2013; Lack et al. 2015; Miller et al. 2016). Our study confirmed that a majority of PHS were mutations accumulated between paralogs. Such PHS due to divergence between paralogs provided a good way to identify segmental duplications. In addition to tandem duplications, low copy–interspersed duplications can also be detected in haploid genomes. However, the breakpoints and locations of interspersed duplications still cannot be defined. We expect that improving long read–sequencing qualities and algorithms will provide better resolution.

The distribution of PHS in genomic regions was uneven; enrichment in coding and 3′UTR regions and depletion in 5′UTR and intron regions. The enrichment of PHS in coding regions might be associated with the accelerated evolution of over one-half of PHS duplications in protein coding regions. The rapid evolution of PHS duplication in coding regions is consistent with the results of other studies in young duplicate protein-coding genes across different organisms (e.g., Lynch and Conery 2000; Kondrashov et al. 2002; Yampolsky and Bouzinier 2010, 2014). The depletion in intronic PHS has not yet been reported elsewhere. It is possible that the depletion is associated with their splicing or cis-regulatory functions, particularly in the first intron which usually plays an important role in transcriptional regulation (Majewski and Ott 2002; Marais et al. 2005). The depletion of 5′UTR PHS is consistent with previous studies which showed 5′UTR partial gene duplications to be less frequent than 3′UTR partial gene duplications (Emerson et al. 2008) and transposable elements to be rarer in 5′UTR than in 3′UTR (Lipatov et al. 2005), suggesting more selective constraints compared with 3′UTR duplications.

The active functional elements associated segmental duplications were more underrepresented than silent functional elements associated duplications. In addition, the reduction levels of the active functional elements associated duplications were correlated with duplication ages, indicating that the duplications at active functional elements were eliminated over time by purifying selection. Among different active functional elements, Pol II and H3K4me3 occur nearby the transcription start sites and the 5′end of the CDS (Pokholok et al. 2005), which overlap with the 5′UTR regions. We indeed observed a similar depletion pattern between duplications at active functional elements and PHS in 5′UTR regions. Stronger selective constraints of active functional elements relative to silent functional elements have been detected in other fly studies. For example, the chromatin landscape comparison between *D. melanogaster* and *Drosophilamiranda* have shown that active chromatin states indicated by active histone marks are highly conserved, whereas repressive chromatin states indicated by silent histone marks are not (Brown and Bachtrog 2014). This conservation difference between active and silent functional elements can also be seen in their relationships with expression levels of stable expressed genes or old genes; active histone marks were correlated with gene expression by regulating both transcriptional level and stability, but silent functional elements showed a weak correlation (P’erez-Lluch et al. 2015; Yu et al. 2017). These results might reflect that the general state of eukaryotic genes is repressed, and transcription is activated by regulatory proteins binding to specific DNA sequences (i.e., cis-regulatory elements) either directly or indirectly (Britten and Davidson 1969; Anthony et al. 2015). Therefore, duplications occurring at active cis-regulatory regions might interfere with gene expression, resulting in impairing the gene regulatory network. Depletion of duplications at active regions by purifying selection might stabilize the gene regulatory network although some duplications could contribute to evolutionary innovation and were driven to fixation by positive selection.

In conclusion, many polymorphic segmental duplications are lineage specific and segregating at low frequencies. The PHS in haploid genomes are mainly contributed by mutations accumulated after duplication. The underrepresentation of duplications at active functional elements is mainly due to purifying selection. Our study on polymorphic duplications reveals that small segmental duplications frequently occurred but were rapidly eliminated by natural selection.

## Materials and Methods

### *Drosophila* Genomic Data

Twenty-eight haploid embryo genomes from DPGP2 (Langley et al. 2011; Pool et al. 2012) and one genome from ZS30 with isogenic chromosomes 2 and 3 (Chong et al. 2013) were used in this study. One haploid embryo genome from the reference strain, *y*; *cn bw*; *sp* (Langley et al. 2011) was used as a control to test the filtering pipeline. The strain numbers, read lengths, insert sizes, and sequencing coverages are summarized in supplementary table S1, Supplementary Material online.

### Read Mapping, Quality Filtering, and PHS Calling

The Illumina FASTQ reads were mapped to the *D. melanogaster* reference genome (BDGP release 5.42). Low-quality parts of all reads were trimmed by trimmomatic-0.22.jar with default parameters (Bolger et al*.* 2014). For comparable read lengths (70–80 bp) and accuracy in mapping small-size duplications, the 146-bp reads were split into two 73-bp fragments. Reads were mapped by BWA version 0.6.1-r104 with default settings (Li and Durbin 2009), and only uniquely mapped reads with mapping quality >30 were used to generate a binary variant call format file of raw variants (both PHS and indels) for each sample by SAMtools Version 0.1.16 and bcftools(1.1) (Li 2011).

We used following criteria to identify the PHS: 1) minimum base quality score of Q25 for both alleles, 2) located at ≥15 bp away from both ends of reads for at least five reads and both alleles with total read depth larger than eight, and 3) not within 6-bp homopolymer, repeat regions or 5-bp extension of an indel. The repeat regions were identified by any 30-bp genomic fragment with more than one hit after remapped to reference genome by bwa with default settings. The indels were determined by the criteria of genotype quality >30 and >5 reads.

### Identification of Duplications

After removing potential sequencing or mapping errors, most PHS were contributed by duplications. To define the cutoff for merging heterozygous sites into a candidate duplication region, we counted the number of duplications by merging linked PHS within 100, 200, 300, 400, 500, and 600 bp and found that there were no significant differences on the number of inferred duplications between 400, 500, and 600 bp (supplementary fig. S9, Supplementary Material online, Wilcoxon sum rank test, *P *>* *0.05). Therefore, we merged linked PHS within 400 bp as one PHS duplication.

In parallel, tandem duplications were discovered by split-read method with Pindel (Ye et al. 2009), which defined the tandem-duplication breakpoints by mapping two fragments of a read to the reference in the opposite order. To avoid bias to number of reads mapped to forward (# +) or reverse strands (# −), S1, (“# +” + 1)* (“#−” + 1), was set to greater than 15.

### Estimation of Copy Numbers by Read Counts

The copy numbers of unique regions across the entire haploid genome were estimated by read-depth method. Reads were split and trimmed to 31–40 bp and mapped to the repeat-masked reference by mrFAST (Alkan et al. 2009). The copy number of each 200 bp was estimated from these mapped short reads by mrCaNaVaR (Alkan et al. 2009). High-copy regions were defined as continuous genomic regions with at least one 200-bp window larger than 2 times and the remaining windows larger than 1.25 times relative to the read-depth mean of the overall genome. If >50% of the length of a PHS duplication overlapped high-copy regions, this PHS duplication was defined as being in high-copy regions.

### Estimation of Copy Numbers by qPCR

Twenty DNA fragments with heterozygous sites were validated by real-time qPCR. All primers were designed by Primer3 (supplementary table S15, Supplementary Material online; Untergasser et al*.* 2012) to produce 60–220-bp amplicons flanking target heterozygous sites. The copy numbers were estimated based on three technical replicates from two of the three strains—FR229, ZS11, and ZS30—using the genome reference strain as a standard to calibrate the ΔΔ*Ct* values. The average *Ct* values of three replicates were calculated after normalization against a single-copy fragment (Livak and Schmittgen 2001). For each amplicon, the copy number was estimated as 2^ΔΔ^^*Ct*^ where ΔΔ*Ct* was normalized as *Z* score*.* Amplicons with estimated copy numbers larger than ∼1.41 (2^0^^.5^) were defined as duplications as described in D’haene et al. (2010). The qPCR reactions were performed using the iQ SYBR Green Supermix (Bio-Rad) on the CFX96 Touch Real-Time PCR Detection System (Bio-Rad) according to the manufacturer’s instructions.

### Identification of Duplicate Homologs in *D. simulans*

To see if any duplication occurred before the *D. melanogaster* and *D. simulans* lineage split, homologs of duplicate regions in the *D. simulans* genome (r2.01; Hu et al. 2013) were identified using nucleotide BLAST (2.2.27+; Altschul et al. 1990). Only the regions with alignment length longer than 50% of the total length and identity larger than 90% were counted as homologs. For comparison, homologs of 2,282 randomly selected 400-bp genomic fragments were also searched by applying the same criteria for 1,000 resamplings.

### Estimating Substitution Rates of Duplications in Protein Coding Regions

To estimate the substitution rates of duplications, we generated ortholog and paralog alignment files for each duplication. Two sets of duplications were analyzed. The first data set was the PHS duplications overlapping tandem duplications (i.e., tandem PHS duplications), whose breakpoints were defined by split-read approach. The CDS within these tandem PHS duplications were used to perform calculation. These tandem PHS duplications could be either partial or complete gene duplications. The second data set was a group of complete gene duplications in which complete CDS were within PHS duplications. The coding region of the longest transcript for each gene overlapping duplications was the target CDS for the estimation of substitution rates. To compare the *d*_N_/*d*_S_ between orthologs and paralogs, we used the alignments of *D. melanogaster* and *D. simulans* reference genomes downloaded from the University of California–Santa Cruz Genome Browser (http://hgdownload.soe.ucsc.edu/goldenPath/dm3/multiz15way/alignments/) for orthologous comparisons and the PHS duplications for paralogous comparisons. The substitution rates between paralogs were estimated from the observed PHS difference between duplicated copies. The ratios of nonsynonymous to synonymous substitutions per site (*d*_N_/*d*_S_) between paralogs and orthologs were calculated by CODEML in the PAML 4.X software (Yang 1997).

### Distribution of Noncoding Functional Elements in Duplications

Duplicate regions were annotated based on gene annotations (r5.42) in FlyBase (http://flybase.org/, Gramates et al. 2017), and *Drosophila* noncoding functional elements downloaded from modENCODE (http://data.modencode.org/, Celniker et al. 2009). In *D. melanogaster*, modENCODE released a large amount of data to map binding loci of active marks, silent marks, and transcription factors across a developmental time course and in multiple cell lines (Roy et al. 2010). We used the data of transcription factor binding sites, histone modification and replacement, and other chromatin binding sites generated by the project of “Regulatory Elements in *Drosophila*” (http://data.modencode.org/? Organism=D.%20melanogaster), because those data sets covered multiple developmental stages more evenly and the histone modification ChIP-chip or ChIP-seq were done in the reference genome strain *y*; *cn bw*; *sp* (supplementary table S11, Supplementary Material online). In this study, Pol II binding sites (19 data sets) and active histone modifications (102 data sets; H3K27Ac, H3K4me1, H3K9Ac, H3K4me3, and H3K36me3; reviewed by Li et al. [2007]) were defined as active marks, whereas insulators (18 data sets; dCTCF, CP190, BEAF-32, Su(Hw), Mod(mdg4), and GAF; N’egre et al. 2010) and silent histone modifications (44 data sets; H3K9me3 and H3K27me3; reviewed by Li et al. [2007]) were defined as silent marks. The others were transcription factors (139 data sets). To examine if duplications occur in noncoding functional element regions randomly, we sampled random genomic fragments from the reference genome 10,000 times and counted the number of duplication occurrence at each functional element data set to determine the expected number. The deviation of the observed duplication number from the expected number for each functional element data set was analyzed by permutation test. The duplications were sorted into six categories and combined into three larger groups in an age-ascending order, including young duplications (singleton tandem duplications without divergence), old single duplications (singleton tandem duplications with divergence and singleton nontandem-duplication PHS duplications), and nonsingletons (nonsingleton tandem duplications without divergence, nonsingleton tandem duplications with divergence, and nonsingleton nontandem-duplication PHS duplication). We found that depletion of duplications seemed to occur at some functional elements. To determine whether depletion of duplications tended to occur at specific functional elements, we used sign test to examine whether the observed numbers of duplicated functional elements tended to be smaller than the randomly selected numbers for each duplication group across all data sets of each functional element. The distribution difference of different duplication groups at specific functional elements mapped from different developmental stages was analyzed by Friedman test followed by multiple comparisons by Wilcoxon signed rank test with Bonferroni correction.

## Supplementary Material

Supplementary data are available at *Genome Biology and Evolution* online.

## Supplementary Material

Supplementary Data
