# Supplementary material for: Small Segmental Duplications in Drosophila—High Rate of Emergence and Elimination
Source: Genome Biol Evol. 2019 Jan 28;11(2):486–96. doi: 10.1093/gbe/evz011 (PMC6380325; doi:10.1093/gbe/evz011)
Supplement: Supplementary Data [file evz011_supp.zip › Supporting information legends.docx]

**Supplementary Materials**

**Fig. S1.** Multiple filtering criteria for pseudoheterozygous site calling. Numbers in parentheses were the numbers of candidate pseudoheterozygous sites filtered from the previous step.

**Fig. S2.** Frequency spectra of (A) pseudoheterozygous sites (PHS), (B) PHS duplications, and (C) tandem duplications. The blue line indicates the expected frequency spectrum estimated by Waterson’s method (1975).

**Fig. S3.** Distribution of pseudoheterozygous sites per 1-kbp non-overlapping window on chromosome arms.

**Fig. S4.** Distribution of distances between two closest heterozygous sites.

**Fig. S5.** Venn diagrams for (A) pseudoheterozygous sites (PHS) and (B) PHS duplications located in different types of duplications. Tandem duplications (TD), duplications called by split-read method; High copy regions (HC), potential duplications called by read-depth method; TD overlapping HC (TD & HC), duplications called by both read-depth and split-read methods; Undetermined (U), PHS not mapped into the above duplications.

**Fig. S6.** Estimation of copy numbers by qPCR. Twenty candidate duplication fragments were tested with three single-copy gene regions: *ACP10, e,* and *y*, as internal control. The copy number of each fragment was estimated by two of three target strains FR229, ZS11, and ZS30 relative to the reference strain *y; cn bw; sp* (*iso-1*). Filled and open symbols represent the fragments validated (*Z* score > 1.41 threshold, indicated by the dotted line) and failed to validate (*Z* < 1.41), respectively. The blue dots are validated as duplications by qPCR but not by *in silico* methods. The red dots are not validated as duplications by qPCR but by *in silico* methods.

**Fig. S7.** Numbers of PHS duplications with divergence per 500-kbp non-overlapping window on chromosome arms.

**Fig. S8.** Plot of numbers of pseudoheterozygous sites (PHS) per bp in tandem duplications with different frequencies in the population. X-axis frequency is the number of genomes with a specific tandem duplication in the 29 genomes sampled. A & C, all tandem duplications; B & D, tandem PHS duplications.

**Fig. S9.** The numbers of regions after merging PHS into duplications for every 100 bp increment. Different lowercase letters above bars indicate statistically significant differences between intervals. Statistical significance was determined by Wilcoxon sum rank test, *P* < 0.05.

**Table S1.** Sample overview

**Table S2.** Large duplicated regions (>28 kb) excluded from this study

**Table S3.** List of pseudoheterozygous sites

**Table S4.** Summary of pseudoheterozygous sites (PHS), PHS duplications, tandem duplications, and high copy regions in each strain

**Table S5.** List of tandem duplications

**Table S6.** Copy number comparison among regions with pseudoheterozygous sites (PHS), tandem duplications (TD), and entire genome

**Table S7.** List of duplications inferred from pseudoheterozygous sites (PHS)

**Table S8.** Numbers and PHS duplications in different frequencies and lengths for each chromosomal arm

**Table S9.** Distribution of pseudoheterozygous sites (PHS), PHS duplications and tandem duplications in genomic regions

**Table S10.** The *d_N_*/*d_S_* analyses of paralogs and orthologs

**Table S11.** Deviation from the random distribution of PHS duplications in non-coding functional element regions

**Table S12.** Statistical summary of duplications in non-coding functional element regions

**Table S13.** Statistical *P* values between duplication groups at different functional elements

**Table S14.** Polymorphism estimates of non-coding functional element regions

**Table S15.** Amplicon regions, sizes, and primers for qPCR validation
